# Ancestry Matters in Decoding Immunity

**DOI:** 10.1111/imcb.70143

**Published:** 2026-06-30

**Authors:** Kesong Wu, Amanda J. Oliver, Zewen Kelvin Tuong

**Affiliations:** ^1^ Ian Frazer Centre for Children's Immunotherapy Research, Child Health Research Centre, Faculty of Health, Medicine and Behavioural Sciences The University of Queensland South Brisbane Queensland Australia; ^2^ Infection and Inflammation Program QIMR Berghofer Medical Research Institute Herston Queensland Australia

## Abstract

The Chinese Immune Multi‐Omics Atlas (CIMA) establishes a population‐scale immune multi‐omics atlas by profiling over 10 million peripheral blood mononuclear cells from 428 Chinese adults using paired scRNA‐seq and scATAC‐seq. This resource mitigates longstanding European ancestry bias in single‐cell genomics and enables cell type resolved regulatory mapping across immune populations. Building on this atlas, CIMA‐CLM integrates chromatin sequence features with single‐cell transcriptomic context to predict chromatin accessibility and assess noncoding variant effects, exemplified in FOXP3^+^ regulatory T cells.

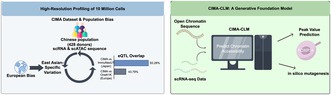

The heterogeneity of the human immune system underpins interindividual differences in disease susceptibility and therapeutic responses. Global immunological research has long been constrained by a significant “Eurocentric” bias; current major single‐cell immune atlases, such as the OneK1K cohort, are predominantly constructed from populations of European ancestry. This representational imbalance creates a critical knowledge gap for non‐European populations, obscuring population‐specific disease and immune mechanisms.

To address this deficiency, Yin et al. [1] present the Chinese Immune Multi‐Omics Atlas (CIMA) (Figure 1). By conducting deep multi‐omics profiling of peripheral blood mononuclear cells (PBMCs) from 428 healthy Chinese adults, the authors established the largest immune single‐cell dataset to date. This resource comprises over 10 million single‐cell transcriptomes and chromatin accessibility profiles. The release of CIMA complements existing projects like Japan's ImmuNexUT [2] and the Human Cell Atlas' Asian Immune Diversity Atlas (AIDA) [3], contributing to a burgeoning foundational resource supporting research into East Asian‐specific immune variation.

**FIGURE 1:**
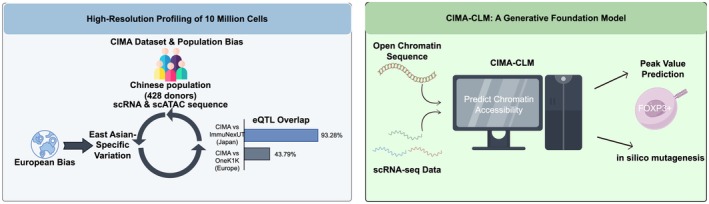
Overview of CIMA. By figdraw.com.

## 1 Counterbalancing Eurocentric‐Bias in Immune Atlases

Although major immune cell lineages in CIMA are broadly conserved across populations and across other large atlases such as European‐based OneK1K and Japanese‐based ImmuNexUT, there were signs that underscored population/ancestry‐specific immune regulatory variations. For instance, expression quantitative trait loci identified in CIMA showed a high degree of concordance with the Japanese cohort ImmuNexUT, with 93.28% overlap, but substantially lower overlap with the European cohort OneK1K at 43.79%.

Beyond regulatory variation at the transcriptomic level, population differences in immune function are also strongly shaped by divergence in the human leukocyte antigen system, which represents one of the most polymorphic regions of the human genome. Large‐scale population genetic studies have documented pronounced differences in HLA allele frequencies and haplotype structures between European and East Asian populations, reflecting distinct demographic histories and pathogen‐driven selective pressures [4].

In this context, CIMA provides a complementary and substantially expanded resource relative to existing Asian‐focused efforts such as the Asian Immune Diversity Atlas (AIDA). AIDA was a multinational single‐cell reference that captures immune diversity across multiple Asian populations and highlights the impact of sub‐continental ancestry on immune cell states and regulatory variants [3]. Together, AIDA and CIMA form synergistic resources, with AIDA emphasizing breadth across Asia and CIMA providing depth and statistical power for fine‐grained immunogenomic dissection.

## 2 A Generative Foundation Model for Multi‐Omics Prediction

Beyond constructing a high‐resolution reference map, the authors also developed a “cell language model” (CLM) for analysis with the CIMA dataset. They devised a method to decode the regulatory genome of cells using the molecular configuration of cells, including cell type or cell state, transcriptional program, chromatin accessibility landscape, and signaling activity.

They utilised cross‐modal integration, where a pretrained genomic model (HyenaDNA) encoded the DNA sequence and a transcriptomic model (scGPT) encoded gene expression, to weigh the influence of distal DNA sequences on gene expression phenotypes effectively.

This architecture enables two transformative capabilities for immunology. First, it achieves zero‐shot prediction. The model can accurately predict the chromatin accessibility landscape (scATAC‐seq) of a cell solely based on its DNA sequence and gene expression. This holds true even for samples it has never seen before.

Second, and most practically, it facilitates “in silico mutagenesis.” Traditionally, determining whether a noncoding variant is functional requires laborious wet‐lab experiments. CIMA‐CLM allows researchers to perform these experiments virtually. Researchers can computationally mutate a nucleotide in the model input and observe the predicted change in chromatin state. This provides a high‐throughput and AI‐driven method to screen millions of variants and prioritize those likely to disrupt immune regulation.

## 3 Uncovering Ancestry‐Specific Regulation and Disease Mechanisms

The ultimate value of a high‐resolution atlas lies in its ability to translate data into biological insight. By integrating the CIMA dataset with genome‐wide association studies (GWAS), the authors demonstrate how ancestry‐specific data could help dissect the molecular etiology of immune‐mediated diseases.

A critical finding of the study involves regulatory variants that are prevalent in East Asian populations but rare in Europeans. These variants often drive distinct immunological traits that would be missed in Eurocentric cohorts. A striking example is the variant rs11886530 in CD4+ naive T cells, which has a frequency of approximately 38% in East Asians compared to only 6% in Europeans. Functional analysis within CIMA reveals that this variant specifically regulates the expression of NPAS2 and NR1D1, core components of the circadian clock machinery. This finding suggests a population‐specific mechanism linking circadian rhythm regulation to immune homeostasis. Circadian influences on immune cell function have been widely documented, including effects on lymphocyte activation, trafficking, and inflammatory responses. In this context, their results raise the possibility that such regulatory links may vary across racial and ethnic groups [5, 6, 7].

Beyond characterizing population frequency, CIMA provides the resolution to map vague genetic risk loci to specific cell types and causal genes. Standard GWAS often implicates noncoding regions without revealing the underlying mechanism. By systematically overlapping GWAS summary statistics with cell‐type‐specific expression quantitative trait loci (eQTLs) and chromatin accessibility quantitative trait loci (caQTLs), the authors identified putative causal variants that simultaneously influence disease risk and local gene regulation. The study focussed on the asthma risk variant rs34415530, which was previously associated with asthma with unclear mechanisms. The authors suggest that regulatory T cells (Tregs) could be a specific driver cell type, as their analysis showed that this variant modulates the expression of Eos (IZKF4), an Ikaros family transcription factor, exclusively in Tregs. Disruption of Eos expression was associated with dysregulated IL‐12B levels, supporting a regulatory relationship in which genetic perturbation of regulatory T‐cell function contributes to inflammatory processes relevant to asthma.

The ability to resolve such cell type‐specific regulatory mechanisms may have important implications for disease biology and target prioritization in the context of east‐Asian populations. This further suggests that current population‐agnostic strategies for discovering novel therapeutic targets may not be adequate; instead, they must be tailored to the specific regulatory failures prevalent in a patient's ancestral background. Such findings reinforce that genomic diversity is fundamental in medicine, and ancestry‐aware drug design holds potential in current gaps.

## 4 Challenges and Future Directions

While CIMA represents a landmark achievement in scale and resolution, integrating large ancestry specific datasets into a global framework remains challenging. A primary difficulty lies in the harmonization of cell type definitions across independently generated atlases. Differences in experimental protocols, sequencing technologies, and laboratory‐specific annotation strategies introduce inconsistencies in how cell types and cell states are defined and labeled, complicating cross atlas comparison and meta‐analytical integration. Such annotation discrepancies have been identified as a fundamental obstacle to large scale atlas integration [8].

In addition, similar to many large‐scale immune profiling efforts, CIMA primarily focuses on peripheral blood, which captures only a subset of immune cell diversity and function. Tissue focused immune atlases generated under consortia such as the Human Cell Atlas and HuBMAP have demonstrated that many immune processes relevant to development, homeostasis, and disease are mediated by tissue resident immune cells and their local cellular environments that are not fully represented in systemic circulation [9]. Consequently, regulatory effects inferred from blood‐based atlases may not fully recapitulate the cellular interactions and molecular programs operating within disease relevant tissues.

Moreover, although reference atlases enable systematic association of genetic variation with cell type‐specific molecular profiles, translating these associations into mechanistic insight requires further validation. Such atlas‐based observations primarily serve as a foundation for hypothesis generation and must be complemented by functional studies to establish causal links between genetic variants, cellular regulatory programs, and disease phenotypes [10].

Recent methodological advances are working toward reconciling some of these challenges. For example, CellHint introduces a principled framework for automated harmonization of cell type annotations by explicitly modeling semantic relationships across datasets, thereby facilitating consistent integration of independently generated atlases [8]. Complementarily, foundation model–based approaches such as SCimilarity demonstrate how a unified metric‐learning representation can enable scalable mapping and querying of new datasets against large, diverse references while preserving fine‐grained biological structure, supporting iterative and extensible atlas integration [11].

Beyond these approaches, emerging artificial intelligence agent‐based systems such as Biomni illustrate the feasibility of autonomously orchestrating complex biomedical analyses across diverse datasets and analytical tools. Although still at an early and exploratory stage, such systems suggest a potential future direction for automating aspects of data integration, annotation reconciliation, and analytical workflow composition at scale. Applying these complementary strategies to integrate CIMA with global immunogenomic resources will enable the construction of a consensus meta‐atlas. Achieving such integration would demonstrate that expanding genomic diversity is not merely an exercise in inclusivity, but a scientific prerequisite for uncovering the full spectrum of human immune regulation and for informing precision therapeutic strategies across populations.

## Author Contributions

**Kesong Wu:** writing – original draft, writing – review and editing, conceptualization. **Amanda J. Oliver:** conceptualization, writing – review and editing. **Zewen Kelvin Tuong:** conceptualization, writing – review and editing.

## Conflicts of Interest

The authors declare no conflicts of interest.

## Data Availability

Data sharing not applicable to this article as no datasets were generated or analysed during the current study.
